# A Time-Course Study on a Food Contact Material (FCM)-Certified Coating Based on Titanium Oxide Deposited onto Aluminum

**DOI:** 10.3390/biology11010097

**Published:** 2022-01-08

**Authors:** Alessandro Di Cerbo, Andrea Mescola, Giuseppe Rosace, Valentina Trovato, Roberto Canton, Ramona Iseppi, Roberta Stocchi, Shakira Ghazanfar, Stefano Rea, Anna Rita Loschi, Carla Sabia

**Affiliations:** 1 School of Biosciences and Veterinary Medicine, University of Camerino, 62024 Matelica, Italy; roberta.stocchi@unicam.it (R.S.); stefano.rea@unicam.it (S.R.); annarita.loschi@unicam.it (A.R.L.); 2 CNR-Nanoscience Institute-S3, 41125 Modena, Italy; andrea.mescola@nano.cnr.it; 3 Department of Engineering and Applied Sciences, University of Bergamo, 24044 Dalmine, Italy; giuseppe.rosace@unibg.it (G.R.); valentina.trovato@unibg.it (V.T.); 4 Moma Nanotech S.r.l., 20861 Brugherio, Italy; canton@nanotech.it; 5 Department of Life Sciences, University of Modena and Reggio Emilia, 41125 Modena, Italy; ramona.iseppi@unimore.it (R.I.); carla.sabia@unimore.it (C.S.); 6 National Agricultural Research Centre, National Institute of Genomics and Agriculture Biotechnology (NIGAB), Park Road, Islamabad 45500, Pakistan; shakira_akmal@yahoo.com

**Keywords:** aluminum, food contact materials, sanitizing treatments, TiO_2_, bacterial inhibition

## Abstract

**Simple Summary:**

In this study, we confirm the bactericidal effect of a special anodizing method, based on TiO_2_ nanoparticles (DURALTI^®^) deposited on aluminum disks with different roughness and subjected to two sanitizing treatments, and, consequently, performing a time-course evaluation against both Gram-negative and Gram-positive bacteria. DURALTI^®^ coating already confirmed its ability to induce a 4-logarithmic decrease (from 10^6^ to 10^2^ CFU/mL) after 6 h. Once each sanitizing treatment was applied, an overall bacterial inhibition occurred in a time ranging from 15′′ to 1′.

**Abstract:**

Aluminum is the second most widely used metal worldwide. It is present as an additive in cosmetics, pharmaceuticals, food, and food contact materials (FCM). In this study, we confirm the bactericidal effect of a special anodizing method, based on TiO_2_ nanoparticles (DURALTI^®^) deposited on aluminum disks with different roughness and subjected to two sanitizing treatments: UV and alcohol 70%. Consequently, we perform a time-course evaluation against both Gram-negative and Gram-positive bacteria to better frame the time required to achieve the best result. Approximately 10^6^ CFU/mL of *Escherichia coli* ATCC 25922; *Salmonella* Typhimurium ATCC 1402; *Yersinia enterocolitica* ATCC 9610; *Pseudomonas aeruginosa* ATCC 27588; *Staphylococcus aureus* ATCC 6538; *Enterococcus faecalis* ATCC 29212; *Bacillus cereus* ATCC 14579 and *Listeria monocytogenes* NCTT 10888 were inoculated onto each aluminum surface and challenged with UV and alcohol 70% at 0, 15”, 30”, 1′, 5′, 15′, 30′, 1, 2, 4 and 6 h. DURALTI^®^ coating already confirmed its ability to induce a 4-logarithmic decrease (from 10^6^ to 10^2^ CFU/mL) after 6 h. Once each sanitizing treatment was applied, an overall bacterial inhibition occurred in a time ranging from 15′′ to 1′. The results are innovative in terms of preventing microbial adhesion and growth in the food industry.

## 1. Introduction

Aluminum is the second most widely used metal worldwide, owing to its castability, plasticity, low melting point and density, heat conductivity and oxidative passivation [1]. It is present as an additive (as itself or derivatives) in cosmetics [1], pharmaceuticals (pH-regulation of heartburn medicine, and vaccine adjuvants) [2], food of both plant and animal origins (vegetables, milk products, sausages and seafood) [3,4,5] as well as food contact materials (FCM) (beverage and food cans, coffee pots, cooking and kitchen utensils, coffee capsules and household aluminum foil) [6,7,8,9].

In 2008, the European Food Safety Authority (EFSA) set the provisional tolerable weekly intake of aluminum to 1 mg kg^−1^ body weight [6], while Regulation (EC) No. 1333/2008 sought to regularize food additive use, including aluminum, along with their maximum level [10]. Moreover, in 2013, the Council of Europe set the maximal amount of metal ions (mg), including aluminum, that can migrate from an FCM surface to the food (kg), i.e., 5.00 mg/kg foodstuff [11]. Nevertheless, recent toxicological studies report that the daily intake of aluminum derived from food ranges from 3.4 to 9 mg/day, thus continuing to pose a serious concern to public health due to an overdosage [1,12]. In fact, several reports highlight the toxic role of aluminum, including, but not limited to, the induction of oxidative stress [13], organ inflammation [14], immunosuppression [13], protein denaturation and transformation [15], apoptosis [13] and endocrine disruption [16].

Nowadays, the use of aluminum and alloys as an FCM is discussed in Regulation (EC)1935/2004, which specifically deals with materials and articles intended to come into contact with food [17].

In addition to aluminum intoxication, another risk for consumers derived from FCM is microbial contamination, and the consequent spoilage of food with repercussions also on health, sensory and nutritional features [18].

In the last decade, nanotechnology offered several opportunities to significantly reduce the release of metal ions, the use of corrosive sanitizing agents and the microbial contamination preserving, at the same time, both food quality and the consumer’s health [18,19,20,21,22,23].

In this sense, many techniques, such as physical vapor deposition, plasma-enhanced chemical vapor deposition, atmospheric-pressure plasma deposition, sol-gel and anodizing, have been proposed to facilitate the deposition of a coating onto FCM, making them antibacterial, non-cytotoxic and easy to clean at the same time [24].

In particular, anodizing is typical of aluminum-based materials that, once placed in an electrolytic cell with aqueous sulfuric acid acting as an electrolyte, form a film of aluminum oxide of 0.08 μm covered by a porous layer of up to 25 μm, according to the equation: 2Al + 3H_2_O → Al_2_O_3_ + 6H^+^ + 6e^−^. To date, nanocomposites (nanoparticles, clay and silicate nanoplatelets, carbon nanotubes, starch nanocrystals and cellulose-based nanofibers) are the most used materials in the food industry, for packaging and coating purposes [25,26,27,28]. Owing to their intrinsic photocatalytic activity or the release of metallic ions, silver [29,30,31], golden [32,33,34] and metal oxide (TiO_2_, ZnO, Fe_3_O_4_, CuO, MgO) [8,35,36,37,38,39] nanoparticles showed the most effective and reliable biocidal activity against both Gram-positive and Gram-negative bacteria via ROS generation [40]. In particular, TiO_2_, a thermally stable, biocompatible, semiconducting transition metal, endowed with good resistance to chemical erosion and a wide bandgap of 3.2 eV, has also been extensively used for other purposes, including deodorant and self-cleaning [41,42,43,44].

In this study, we aimed to confirm the bactericidal effect of a special anodizing method based on TiO_2_ nanoparticles deposited onto aluminum disks with different roughness and subjected to two sanitizing treatments, and, consequently, performing a time-course evaluation to better frame the time required to achieve the best result.

## 2. Materials and Methods

### 2.1. Samples and Coating

Four hundred and thirty-two round-shaped aluminum disks with a 5 cm diameter, 0.5 cm thickness and three different surface roughness, (R_a_) 0.25 ± 0.02 μm (R0.25, *n* = 144), 0.5 ± 0.03 μm (R0.5, *n* = 144) and 1 ± 0.06 μm (R1, *n* = 144), were used in this study. A total of 216 disks were coated with a special anodizing based on Al_2_O_3_–TiO_2_, named DURALTI^®^ [45], approved for food contact according to the FCM certification, while the remaining 216 disks were left uncoated. DURALTI^®^ is characterized by a hardness of 500 HV, a low abrasion index (<10 mg of weight loss) according to the ASTM D 4060 standard [46], a static friction coefficient of 0.2, and a dynamic friction coefficient of 0.15, as well as strong resistance corrosion (almost 1000 h) according to the ASTM B 117 standard [47]. Furthermore, it is compliant with Regulation (EC)1935/2004 [17], and with DIN 10,531 [48] and UNI 11,460 [49] standards for the lack of lead and nickel release in the production and dispense of hot beverages from hot beverage appliances [48].

### 2.2. Microbiological Analysis

Gram-negative (*Escherichia coli* ATCC 25922, *Salmonella* Typhimurium ATCC 1402, *Yersinia enterocolitica* ATCC 9610, and *Pseudomonas aeruginosa* ATCC 27588) and Gram-positive (*Staphylococcus aureus* ATCC 6538, *Enterococcus faecalis* ATCC 29212, *Bacillus cereus* ATCC 14579 and *Listeria monocytogenes* NCTT 10888) strains were purchased from LGC Standards S.r.l. (Sesto San Giovanni, Milan, Italy). Subsequently, they were grown in tryptic soy broth (TSB, bioMérieux, Florence, Italy), incubated at 37 °C for 24 h and activated by two successive transfers.

### 2.3. Inoculum Preparation

For each strain, 100 μL of the overnight culture was transferred into 10 mL of tryptic soy broth (TSB, Oxoid, Milan, Italy) and incubated at 37 °C with an oscillating speed of 150 rpm. After 5 h, the optical density (OD) was determined at 600 nm using a microtiter plate reader (Sunrise™, Tecan Trading AG, Männedorf, Switzerland), and the viable cell count was determined by spreading on tryptic soy agar (TSA, Oxoid, Milan, Italy) plates. Approximately 10^6^ CFU/mL of each strain was inoculated onto each aluminum surface, according to the procedure previously described [21].

### 2.4. FTIR Characterization

Both uncoated and DURALTI^®^-coated aluminum disks were characterized by Fourier transform infrared spectroscopy (FTIR). FTIR spectra were recorded with a Thermo Avatar 370, equipped with an attenuated total reflection (ATR) device using a diamond crystal as an internal reflectance element. Spectra were acquired at room temperature in the range from 4000 to 530 cm^−1^, with 32 scans and a resolution of 4 cm^−1^.

### 2.5. Time-Course Assay, Sanitizing Procedures and Surface Swabbing

A total of 27 coated and 27 uncoated disks were used for each bacterial strain: for each roughness, 9 coated and 9 uncoated disks were used for control (*n* = 3), UV (UVC, 253 nm, *n* = 3) and alcohol 70% (*n* = 3) treatment.

Each aluminum surface was swabbed 10 times vertically and horizontally, using a moistened sterile cotton (Incofar, Modena, Italy) at different times (0, 15”, 30”, 1′, 5′, 15′, 30′, 1, 2, 4 and 6 h).

Then, the cotton head was aseptically broken off and placed in a tube with 1 mL of sterile saline 0.9% solution and vortexed for 1′. Serial tenfold dilutions were spread onto selective agar plates and the viable cells were counted after incubation at 37° C for 24 h.

### 2.6. Environmental Scanning Microscopy Analysis (ESEM)

An ESEM Quanta-200 (ThermoFisher Scientific, Rodano, Italy) connected with a microanalysis system X-EDS Oxford INCA-350 was used to obtain micrographs and spectra of each roughness, both on coated and uncoated aluminum disks.

Each sample was mounted on an aluminum stub via double-sided adhesive tape and observed without sputtering at a high vacuum (≈10^−5^ Torr), with an accelerating voltage of 25 kV, working distance between 11 and 12 mm, spot size 4 µm and standard acquisition resolution 1024 × 943 pixels.

Micrographs at 500× original magnifications were acquired while sample areas of ~300 μm × 300 μm were being investigated.

### 2.7. Contact Angle (CA) Measurements

Static CA values of uncoated and DURALTI^®^-coated aluminum disks were measured by a homemade instrument equipped with a high-speed CCD camera.

The used equipment allows the determination of CA, with a precision of ±1°, by taking images at frequencies as high as 200 Hz, starting within a few tens of milliseconds after the deposition of the drop. All measurements were performed under an ambient atmosphere at room temperature and common moisture of RH = 40 ± 5%. Double-distilled water with a conductivity less than 10^−6^ S · m^−1^ at 25 °C was used for cleaning all materials and contact angle measurements. Before all measurements, uncoated and coated samples were cleaned with such a water, thoroughly rinsed with ethanol to minimize any potential surface contamination, and finally dried for 24 h at least. A drop of high purity distilled water was located on the surface of the samples, and the image of the droplet was recorded after 10′′ with the CCD camera. The drop volume was chosen within a range for which the CA did not change with the variation of the volume (4 ± 0.5 μL).

The CA values were determined using an image analysis software (ImageJ 1.46r, Wayne Rasband, National Institutes of Health, Bethesda, Maryland, USA). According to other research findings that confirmed the close correlation between the surface topography and the measured angles, the final CA values were obtained by averaging measured data at ten randomly selected points [50,51]. For each sample with the same surfaces roughness, the CA change (*CA_change_*) between uncoated and coated disks was obtained by the following Equation (1):

(1) $$CA_{change}\left(\%\right)=\frac{CA_{coated}-CA_{uncoated}}{CA_{uncoated}}\;\times \;100$$

where *CA_coated_* is the CA of the coated surface and *CA_uncoated_* is the CA of the uncoated surface.

## 3. Results

### 3.1. FTIR Characterization

**Figure 1:**
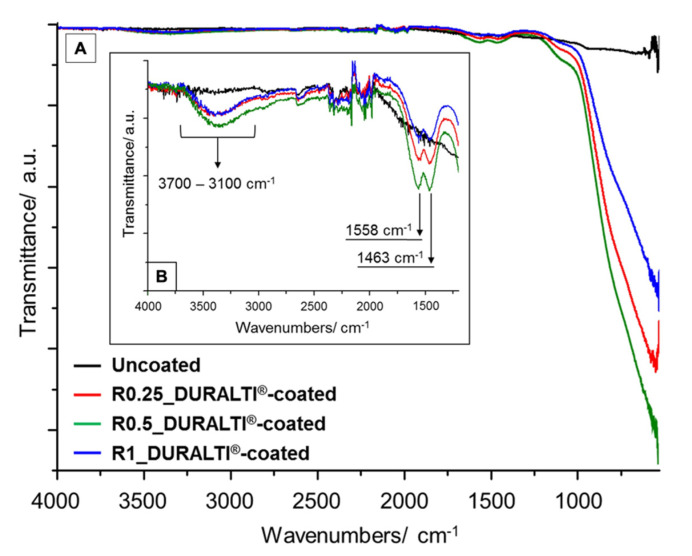
FTIR spectra of R0.25, R0.5 and R1 uncoated and DURALTI^®^-coated aluminum disks in the range between (**A**) 4000–530 cm^−1^ and 4000–1200 cm^−1^ (**B**).

FTIR spectra of uncoated and DURALTI^®^-coated aluminum disks with different roughness were investigated in the range 4000–530 cm^−1^ (Figure 1A). To improve readability, FTIR spectra are highlighted in the range 4000–1200 cm^−1^ (Figure 1B). In Figure 1, the major infrared absorption features at 3700–3100, 1558 and 1463 cm^−1^ are assigned to AlO-H stretching modes on the outer geometrical surface of the Al_2_O_3_ [52], and to oxalate skeleton vibrations (C–O stretching and C–H bending) as a residue of the applied precursor, respectively [53].

Compared to the uncoated sample, the infrared spectra of coated aluminum disks show a strong and very broad band above 1180 cm^−1^ (Figure 1A), which is attributed to (i) asymmetric and symmetric bending modes of Al–O–Al bonds [54,55]; (ii) stretching vibrations of Al–O bonds [56] and(iii) Ti–O–Ti, Al–O–Al and hetero metal–oxygen Ti–O–Al bonds [54,57].

### 3.2. Time-Course Assay

**Figure 2:**
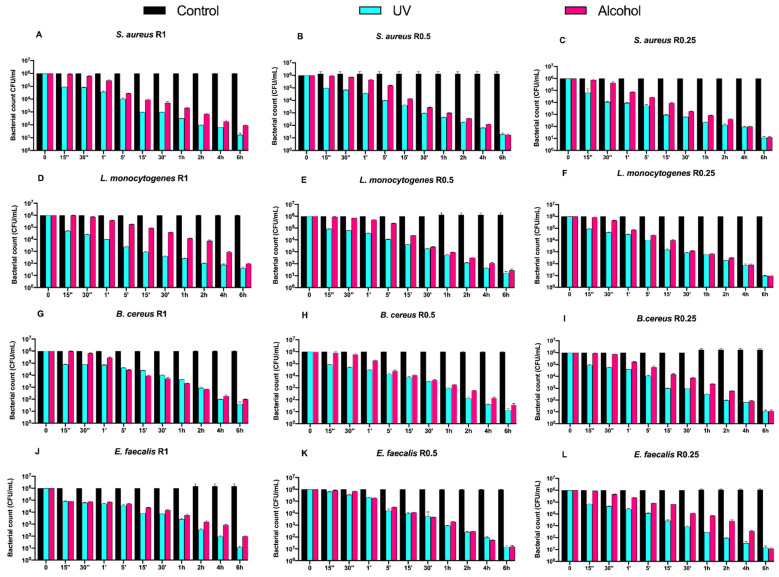
Graphical representation of Gram-positive bacterial counts (**A**–**L**) at different exposure times on uncoated aluminum disks with different surface roughness (R0.25, R0.5 and R1 μm) and subjected to UV and alcohol sanitizing methods.

Counts (CFU/mL) of Gram-positive bacteria, at different exposure times on uncoated aluminum disks with 3 different types of roughness, subjected to UV and alcohol sanitizing methods, are reported in Figure 2.

Regardless of the roughness and exposure time, no visible microbial count decrease was observed for all control disks. Both alcohol and UV treatments induced a time-dependent microbial count decrease down to 5 logarithms (from 10^6^ to 10^1^ CFU/mL) after 6 h of exposure, with a slower trend for the former (Figure 2A–L).

**Figure 3:**
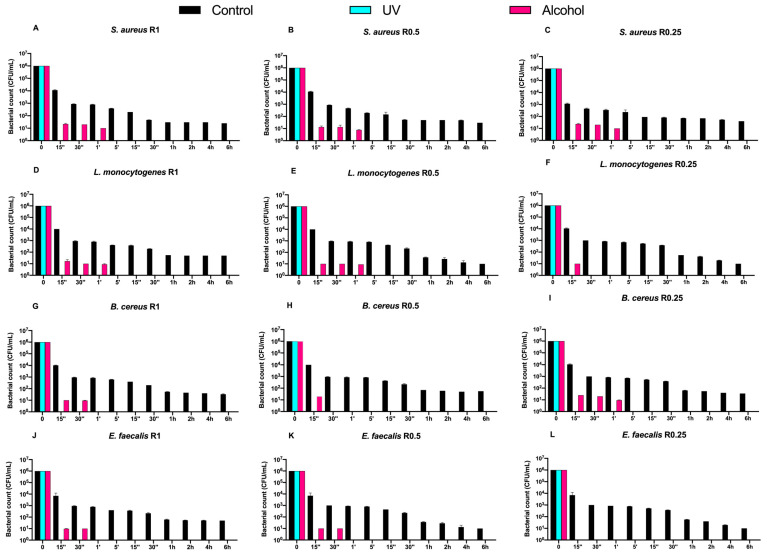
Graphical representation of Gram-positive bacterial (**A**–**L**) counts at different exposure times on DURALTI^®^-coated aluminum disks with different surface roughness (R0.25, R0.5 and R1 μm) and subjected to UV and alcohol sanitizing methods.

Then, we also evaluated the time-course counts of the same Gram-positive bacteria on DURALTI^®^-coated disks (Figure 3).

Dealing with the controls, a 4-logarithmic decrease (from 10^6^ to 10^2^ CFU/mL) was observed on the coated disks after 6 h, regardless of roughness. Moreover, an overall bacterial growth inhibition was already observed for all strains before 15′′ of UV exposure regardless of roughness, while a slightly slower trend was observed for strains subjected to alcohol sanitization.

For instance, *Staphylococcus aureus* ATCC 6538 was already inhibited after 1′ of alcohol exposure regardless of roughness (Figure 3A–C), while *Listeria monocytogenes* NCTT 10888 was inhibited after 1′ only at R1 and R0.5, and after 15′′ at R0.25 (Figure 3D,E). Further, *Bacillus cereus* ATCC 14579 was inhibited at different times of alcohol exposure depending on roughness, in particular after 30′′ and 15′′ and 1′ at R1, R0.5, and R0.25, respectively (Figure 3G–I). Conversely, *Enterococcus faecalis* ATCC 29212 was equally inhibited after 30′′ of alcohol exposure at R1 and R0.5, and after 15′′ at R0.05 (Figure 3J–L).

**Figure 4:**
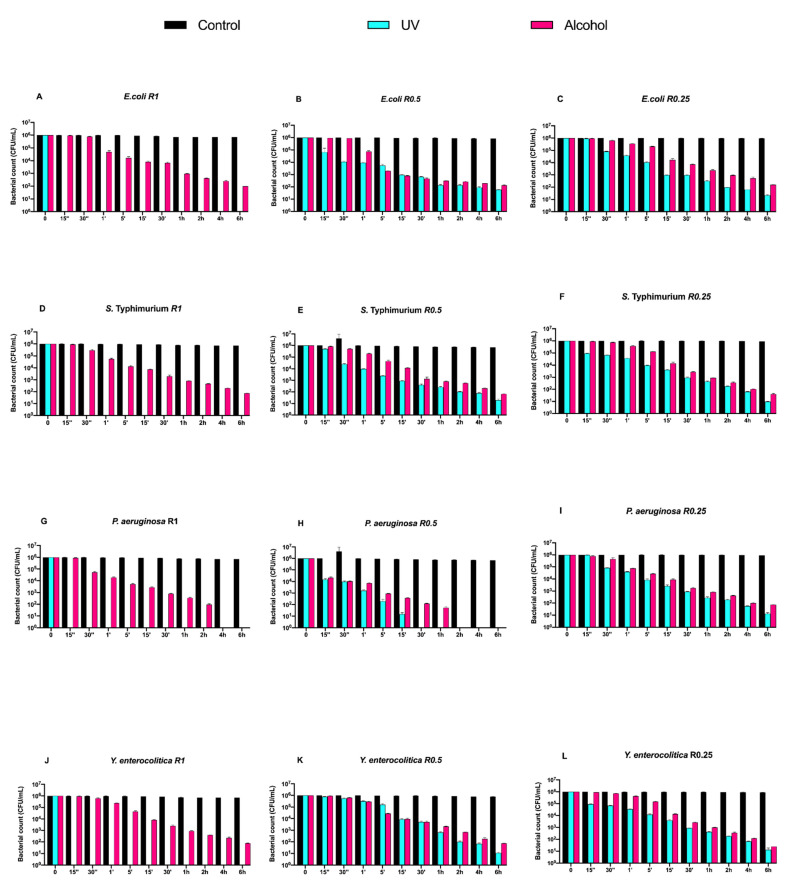
Graphical representation of Gram-negative bacterial counts (**A**–**L**) at different exposure times on uncoated aluminum disks with different surface roughness (R0.25, R0.5 and R1 μm) and subjected to UV and alcohol sanitizing methods.

**Figure 5:**
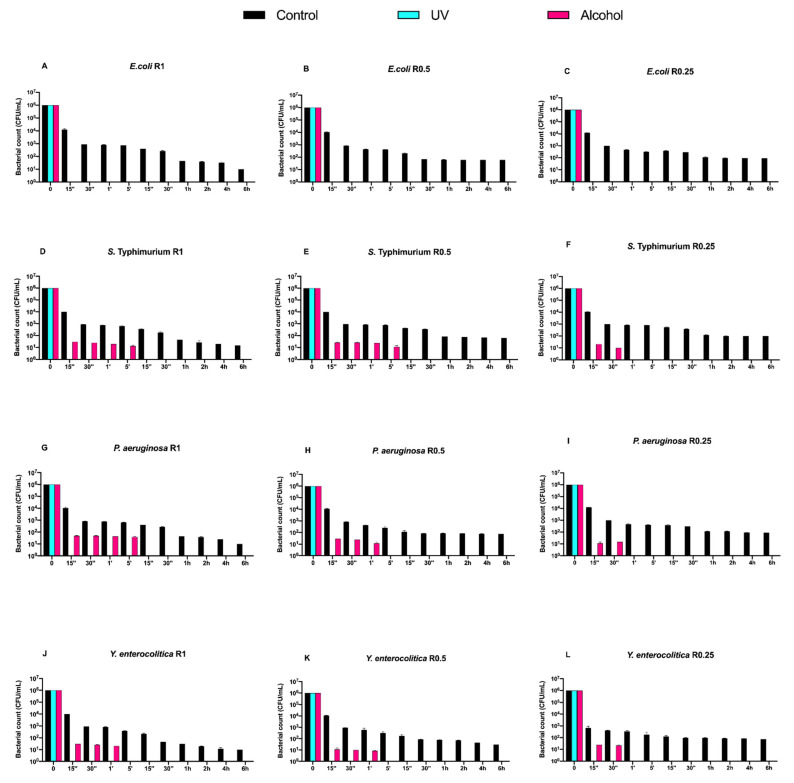
Graphical representation of Gram-negative bacterial counts (**A**–**L**) at different exposure times on DURALTI^®^-coated aluminum disks with different surface roughness (R0.25, R0.5 and R1 μm) and subjected to UV and alcohol sanitizing methods.

Subsequently, we evaluated the growth trend of Gram-negative bacteria on both uncoated and DURALTI^®^-coated disks (Figure 4 and Figure 5).

As observed for Gram-positive bacteria, the uncoated surface did not decrease the Gram-negative load regardless of roughness and strain (Figure 4A–L). In a manner different to Gram-positive bacteria, UV treatment was already able to completely inhibit *Salmonella* Typhimurium ATCC 1402, *Yersinia enterocolitica* ATCC 9610 and *Pseudomonas aeruginosa* ATCC 27588 before 15′′, only at R1 (Figure 3A,D,G).

Conversely, alcohol treatment resulted in an overall 4-logarithmic bacterial growth reduction (from 10^6^ to 10^2^ CFU/mL) after 6 h, with the only exception of *Pseudomonas aeruginosa* ATCC 27,588 at R1 and R0.5, where such a reduction was achieved after 2 h and 1 h, respectively (Figure 4 G,H).

Then, we evaluated the effect of the DURALTI^®^ coating on the same strains, roughness and treatments (Figure 5).

An overall microbial growth reduction (from 10^6^ to 10^2^ CFU/mL) after a 6 h exposure was observed in control samples, with the only exception of all strains at R1, for which a 10^1^ CFU/mL concentration was achieved (Figure 5A–L). Moreover, UV treatment resulted in a complete microbial count decrease in all strains already before 15′′, as observed for Gram-positive bacteria.

Different trends were observed after the alcohol treatment. Dealing with *Escherichia coli* ATCC 25922, a complete inhibition was already achieved before 15′′, regardless of roughness (Figure 5A–C). On the other hand, *Salmonella* Typhimurium ATCC 1402 was completely inhibited after a 5′ at R1 and R0.5, and after a 30′′ at R0.25 (Figure 5D–F). A 5′ exposure was also sufficient to completely inhibit *Pseudomonas aeruginosa* ATCC 27588 at R1, while such a result was achieved after 1′ and 30′′ at R0.5 and R0.25, respectively (Figure 5G–I). *Yersinia enterocolitica* ATCC 9610 was inhibited after 1′ at R1 and R0.5, and after 30′′ at R0.25 (Figure 5J–L).

### 3.3. Environmental Scanning Microscopy Analysis (ESEM)

**Figure 6:**
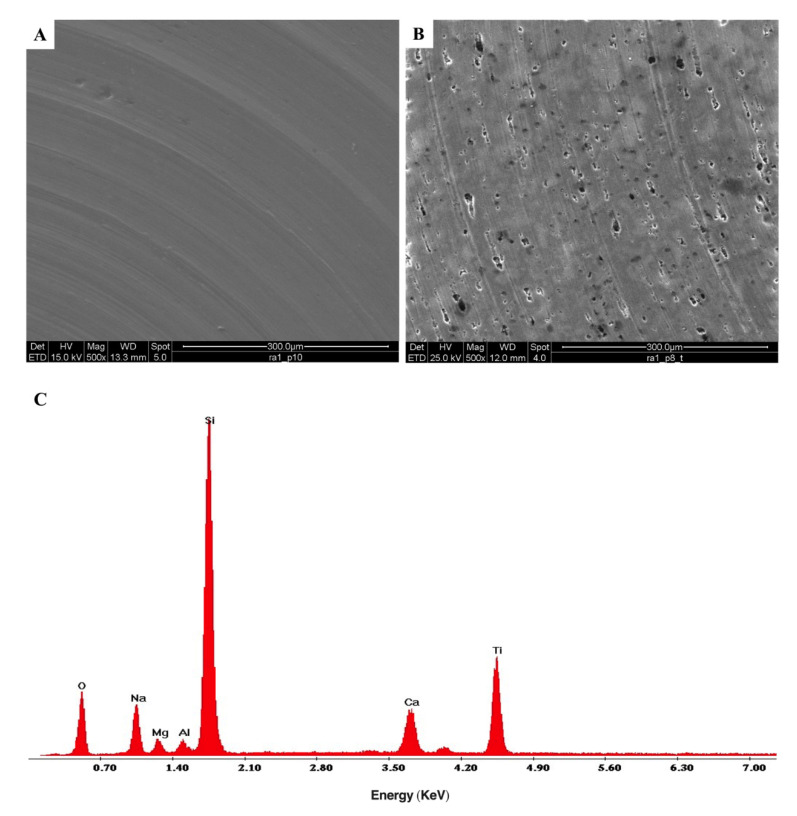
ESEM morphological analysis of (**A**) uncoated, (**B**) DURALTI^®^-coated aluminum disks at 1 μm roughness; (**C**) X-EDS microanalysis of a DURALTI^®^-coated aluminum disk at 1 μm roughness.

ESEM micrographs of both uncoated and DURALTI^®^-coated aluminum disks are reported in Figure 6.

The micrograph of the uncoated aluminum disk clearly shows typical signs of the turning process (Figure 6A), with the characteristic parallel grooves resulting from the gradual removal of surface layers. Instead, the micrograph of DURALTI^®^-coated aluminum disk reveals a heterogeneous structure with the presence of clusters and pores (Figure 6B). Moreover, the X-EDS spectrum of the DURALTI^®^-coated aluminum disk confirmed the presence of TiO_2_ (Figure 6C).

### 3.4. CA and Wettability

**Figure 7:**
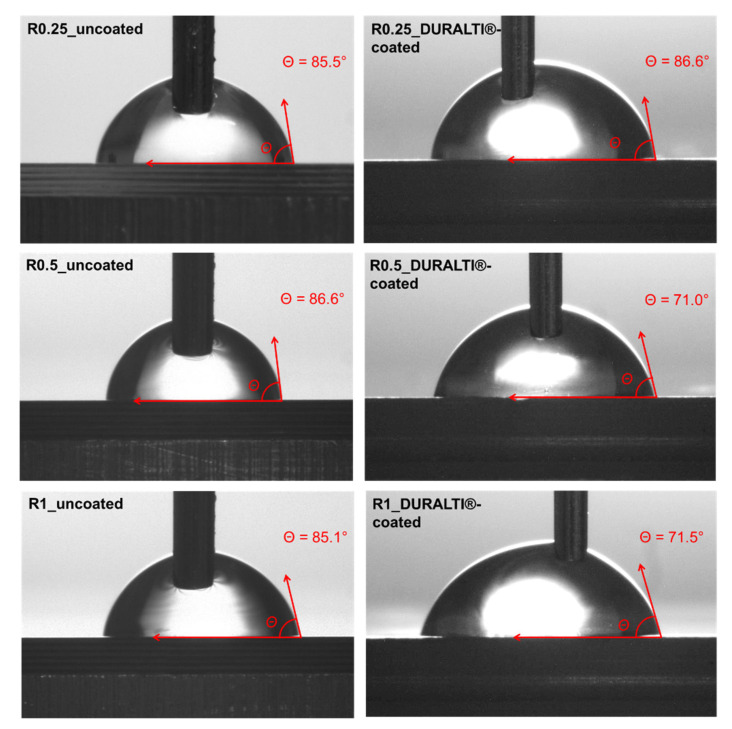
Images of steady-state water CA of uncoated and DURALTI^®^-coated aluminum disks.

The surface wettability of the DURALTI^®^-coated aluminum samples with the 3 different roughness, was investigated by steady-state water CA measurements. Images and CA values of both uncoated and coated aluminum disks are shown in Figure 7 and listed in Table 1, respectively.

The CA percentage change between uncoated and coated samples (Equation (1)) was calculated for each sample as an index of roughness influence on coating wettability: the greater the difference in the negative percentage change in the CA, the more hydrophilic the surface.

As shown in Figure 7 and Table 1, the surface roughness of uncoated samples does not significantly affect the CA values that remain almost constant at around 85–86°. Similar behavior was observed for coated samples at R0.25, where the water drop placed on the surface led to a negligible hydrophilicity effect. Conversely, the surface wettability of the coated samples slightly increases at R0.5 and R1. Indeed, the analysis of the CA change showed that the greatest differences were observed at R0.5 (−18.0%) and R1 (−16.0%), while the smallest difference was observed at R0.25 (1.3%).

## 4. Discussion

### 4.1. Time-Course Assay

Nowadays, aluminum is a ubiquitous material present in many foodstuffs that, in turn, become its main exposure source for humans. Despite the daily intake of 1.7 to 13 mg of aluminum that has been set by EFSA for a 60 kg adult, the untoward release from food contact materials, such as aluminum foils, trays, cans, cookware, utensils and food packaging, cannot be neglected [6,7]. It is therefore fundamental to find reliable solutions to prevent such an untoward release and, at the same time, exert a rapid bacteriostatic and/or bactericidal effect. In this regard, nanotechnological coatings have shown great potential in terms of biocompatibility and leaching prevention [19,20,21,35].

The aim of this study was to provide more details concerning the time required by an FCM-certified coating (DURALTI^®^) deposited on aluminum disks with different roughness, to exert its bacteriostatic/bactericidal effect against different Gram-negative and Gram-positive bacterial strains.

In fact, in a previous study, we observed that, after 12 h of exposure of the same bacteria, a partial bactericidal effect (from 10^6^ to 10^2^ CFU/mL) occurred on DURALTI^®^-coated surfaces without any sanitizing treatment (UVC and alcohol) and regardless of roughness, while a complete inhibition of bacterial growth occurred when each of the sanitizing treatments was applied [21].

We, therefore, carried out a time-course (0–6 h) evaluation to possibly observe the aforementioned effects before 12 h, and to better frame the time required to achieve the most effective inhibition.

Interestingly, DURALTI^®^-coated aluminum disks decreased both Gram-positive and Gram-negative bacterial loads from 10^6^ to 10^2^ CFU/mL already after 6 h, regardless of roughness. On the other hand, both Gram-positive and Gram-negative results were completely inhibited in a time ranging from 15′′ to 1′, once each sanitizing treatment was applied.

As for the uncoated disks, a different trend was observed for Gram-positive and Gram-negative bacteria. A bacteriostatic trend was observed for the controls, regardless of roughness and treatment, and a bactericidal one when treatments were applied, never reaching a complete removal of the bacteria within 6 h. This observation was in agreement with our previous work, in which such a trend was kept up to 12 h [21], thus confirming the hypothesis of a strong binding possibly occurring at the bacteria/surface interface. Although many mechanisms have been proposed to explain such binding (thermodynamic theory, Lifshitz-van der Waals, and electrostatic-double layer interactions, ionic strength and pH) [20,21,58], several studies also demonstrated a direct correlation between bacterial adhesion and surface roughness increase above 0.2 µm [59,60,61,62]. Moreover, despite the trend of controls and treatments of Gram-negative bacteria resulting in more or less the same Gram-positive strains, it is worth noting that a complete removal of the bacteria occurred after 15′′ and 15′ of UVC exposure at R1 for all strains and at R0.5 for *Pseudomonas aeruginosa* ATCC 27588, respectively. Interestingly, *Pseudomonas aeruginosa* ATCC 27588 was also completely inhibited by alcohol after 1 h at R1, and after 2 h at R0.5. Even in this case, the synergistic effect of UVC treatment and surface roughness was in agreement with our previous study, in which a complete bacterial growth inhibition after 12 h of exposure with UVC at R1 was observed [21]. In this sense, some studies evidenced how bacterial adhesion and biofilm formation in some cases can also be negatively influenced by higher surface roughness, in particular for *Pseudomonas aeruginosa* [63,64], despite the fact that they are often positively correlated with such higher roughness.

### 4.2. FTIR Characterization

FTIR characterization, CA measurements and ESEM analysis confirmed the successful deposition of the Al_2_O_3_-TiO_2_ thin film on DURALTI^®^-coated disks.

In particular, ATR-FTIR spectroscopy is a powerful tool for investigating the functional groups present on the surface of treated materials, due to its sensitivity and penetration depth of only a few micrometers.

Since crystallinity degree, as well as morphology and particle size, can affect the IR bands’ position and intensity, such peaks can be correlated with the phase evolution in titania–alumina composites [54]. Therefore, the prominent composition features of the coating can be characterized by absorption bands assigned to vibrations of functional groups typical of the applied coating. Indeed, compared to the reference aluminum sample, the infrared spectra of DURALTI^®^-coated disks revealed characteristic absorption peaks assigned to bending modes of Al–O–Al bonds [54,55], and to stretching vibrations of AlO–H [52], Al–O [56], Ti–O–Ti, Al–O–Al and –Ti–O–Al– bonds [54,57], confirming the total coverage of pristine aluminum surfaces by Al_2_O_3_-TiO_2_ coating. Furthermore, the deposition of Al_2_O_3_-TiO_2_ coating led to changes in aluminum disk wettability, as demonstrated by the CA values. As reported in literature [65], the chemical composition, surface morphology and roughness affect the CA value, according to the Wenzel model [66]. Thus, a lowering in the CA reflects an increase in the surface tension and, consequently, an improvement in the surface hydrophilicity.

In this study, CA measurements of Al_2_O_3_-TiO_2_-coated aluminum disks demonstrated enhanced hydrophilicity compared to uncoated samples, caused by the presence of polar chemical groups, which increased the surface energy of coated samples. In particular, experimental findings suggested that surface roughness values over 0.5 μm equally affect the Al_2_O_3_-TiO_2_ coating morphologies, thus further influencing the static CA. Indeed, above such roughness, the porosity of the applied coating does not improve water droplet absorption. This behavior demonstrates the enhancement of the hydrophilic properties of Al_2_O_3_-TiO_2_ coating applied on aluminum disks in agreement with the performance reported for a similar coating [67,68].

### 4.3. ESEM Analysis

ESEM micrographs carried out on DURALTI^®^-coated surfaces show the presence of a heterogeneous structure, reasonably related to the presence of porous anodized coating.

As proposed by Thukkaram et al. [69], such heterogeneity in the surface morphology can trigger the release of TiO_2_ nanoparticles and the resulting activation of their intrinsic antibacterial properties. Such properties occur as a consequence of further exposure to UVC, promoting free radical release and leading to the killing of pathogenic bacteria [35].

The capability of tuning Al_2_O_3_ porosity and shaping has been thoroughly investigated over the last decades, paving the way to its use in a wide range of fields, including FCM.

## 5. Conclusions

The DURALTI^®^ coating confirmed its ability to induce a 4-logarithmic decrease (from 10^6^ to 10^2^ CFU/mL) against both Gram-negative and Gram-positive bacteria regardless of roughness, but we demonstrated that a 12 h exposure is overestimated since such a decrease already occurs after 6 h.

Moreover, we highlighted that all bacteria were completely inhibited in a time ranging from 15′′ to 1′, after the application of each sanitizing treatment.

Despite some study limitations, such as the lack of a biofilm assay on uncoated and DURALTI^®^-coated surfaces, we believe that the results are innovative since this is one of the first reports dealing with a coating approved for contact with food, and endowed with a self-antibacterial activity. This is important in light of the growing awareness of the potential of nanotechnologically-treated FCM as a new frontier in the prevention of microbial adhesion and growth in the food industry, as well as in the reduction of the abuse of corrosive and toxic sanitizing agents.

## Figures and Tables

**Table 1:** Roughness profile and CA measurements of uncoated and coated aluminum disks. The standard deviation was computed considering ten repeated tests performed on each sample.

Sample	Roughness (µm)	CA (°)	CA Change (%)
R0.25 uncoated	0.25	85.4 ± 4.2°	1.3
R0.25 DURALTI^®^-coated	0.25	86.7 ± 1.5°	
R0.5 uncoated	0.50	86.7 ± 2.1°	−18.0
R0.5 DURALTI^®^-coated	0.50	71.1 ± 6.0°	
R1 uncoated	1.00	85.0 ± 1.3°	−16.0
R1 DURALTI^®^-coated	1.00	71.4 ± 5.4°	

## Data Availability

The data presented in this study are available on request from the corresponding authors.
